# The effects of obesogenic diet on a *Drosophila* model of frontotemporal dementia

**DOI:** 10.17912/micropub.biology.002150

**Published:** 2026-08-19

**Authors:** Charlie Ake, Eric Cervantes, Christal Garcia, Hannah Hammons, Lisa Obawole, Isabella Morton, Lexie Pham, Brandon Salas, Sadie Stacks, Luis Villarreal, Faith York, Samaneh Biglari, Eshani Yeragi, Elizabeth Lillvis, Matthew Moulton, Alex Keene

**Affiliations:** 1 Biology, Texas A&M University, College Station, TX, US

## Abstract

Tau aggregation is a hallmark of neurodegenerative diseases including Alzheimer’s disease and frontotemporal dementia. Here, we tested how an obesogenic diet affects a Drosophila tauopathy model across multiple behavioral assays. In group-housed flies, mutant tau shortened lifespan, and this effect was worsened by a high-sugar diet. We also examined sleep, motor behavior, and sensory responsiveness. Tau-expressing flies differed from controls in all assays, but these deficits were not further exacerbated by diet. These findings reveal assay-specific diet effects and highlight the value of using multiple behavioral measures to characterize tau mutant phenotypes.

**Figure 1. The effects of Tau overexpression on behavior and longevity:** The effects of Tau overexpression on behavior and longevity. A) Survival curves for flies maintained on standard or obesogenic diet under grouped-housed conditions. There was a significant effect of genotype and dietary condition on survival (χ² = 58.55, df = 5, p < 0.0001). Longevity of Tau ^R406W^ flies under high-sucrose conditions was reduced compared to flies housed on a standard diet (χ² = 11.01, df = 1, p < 0.01), while no significant differences were observed in Tau ^WT^ (χ² = 0.01079, df = 1, p > 0.05) or control flies housed on HSD or standard diet (χ² = 0.01070, df = 1, p > 0.05). B) Survival curves collected in individually housed flies in *Drosophila* activity monitors (Log-rank/Mantel–Cox: χ² = 196.7, df = 5, p < 0.0001), with a significant effect across conditions. Longevity was reduced in control flies on sucrose compared to standard diet (χ² = 55.65, p < 0.0001), no significant dietary effect in Tau ^WT^ (χ² = 2.464, p > 0.05), and markedly reduced survival in Tau ^R406W^ (χ² = 6.733, p < 0.05) flies. Tau ^R406W^ flies exhibited reduced survival under both conditions. C) Sleep profiles across the lifespan collected in *Drosophila* activity monitors (Sample size for individually housed flies nSyb-GAL4>+ (n = 40 Std, n = 24 Sucrose), nSyb-GAL4>Tau ^WT^ (n = 48 Std, n = 16 Sucrose), and nSyb-GAL4>Tau ^R406W^ (n = 45 Std, n = 53 Sucrose)). D) Total sleep quantified at Day 14 under standard and obesogenic diets. Two-way ANOVA revealed a significant effect of genotype/diet (F(5, 621) = 203.3, p < 0.0001). Sleep was reduced in nSyb-GAL4/+control flies on HSD compared to standard diet (mean difference = 324.8, p < 0.0001), while Tau ^WT^ , and Tau ^R406W^ flies exhibited significantly higher sleep compared to controls under both conditions (p < 0.0001). No significant difference was observed between diets within Tau ^WT^ , and Tau ^R406W^ groups. (Sample size: nSyb-GAL4>+ (n = 40 Std, n = 24 Sucrose), nSyb-GAL4>Tau ^WT^ (n = 48 Std, n = 16 Sucrose), and nSyb-GAL4>Tau ^R406W^ (n = 45 Std, n = 53 Sucrose)) E) **.** Climbing assay revealed significant effects of genotype and dietary condition (F (5, 424) = 63.67, p < 0.0001). Climbing was significantly reduced in Tau ^R406W^ flies compared to control and Tau ^WT^ group under both dietary conditions (p < 0.0001), while HSD reduced performance in Tau ^WT^ flies and increased performance in Tau ^R406W^ flies. The data are presented as mean ± SE. F) PER comparison in flies on HSD and standard diet revealed a significant effect of diet on PER (F _1, 203_ = 6.91, p = 9.19E-3). PER is decreased in Tau ^R406W^ group under standard (p < 0.0001) and HSD (p < 0.001) compared to control group. nSyb-GAL4>+ (n = 27), nSyb-GAL4>Tau ^WT^ (n = 21), nSyb-GAL4>Tau ^R406W^ (n = 36), nSyb-GAL4>+ fed HSD (n = 138), nSyb-GAL4>Tau ^WT^ fed HSD (n = 56), nSyb-GAL4>Tau ^R406W^ fed HSD (n = 69). * denotes *p < 0.05; **p < 0.01, ***p < 0.001; ****p < 0.0001.

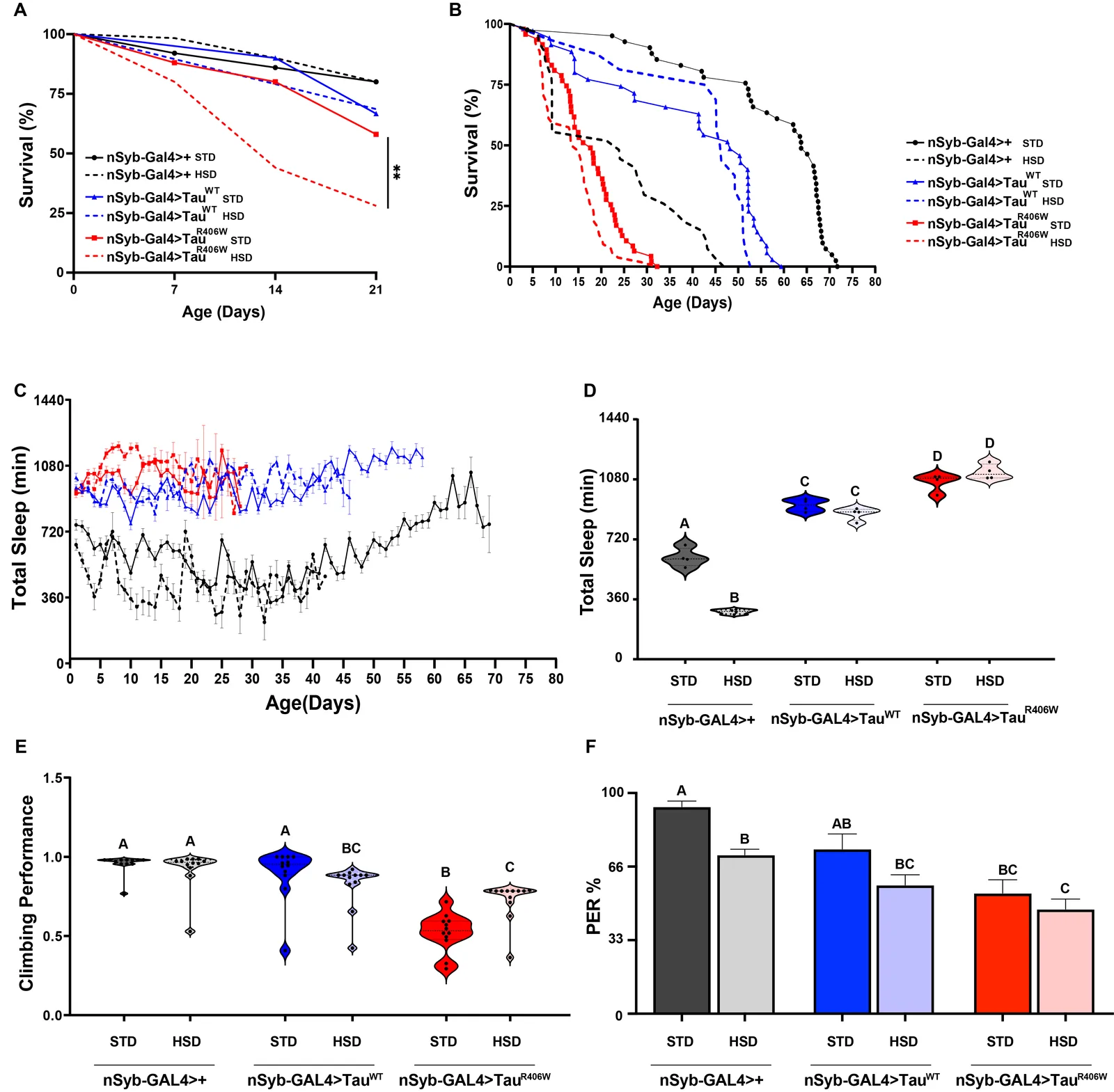



## Description

Large-scale genomic analyses have identified numerous genetic factors that contribute to the pathogenesis of neurodegenerative disease. Studies have also revealed connections between neurodegeneration and life-history traits such as stress, diet, and sleep. The microtubule-associated protein Tau plays a central role in neurodegeneration. Mutations that promote Tau hyperphosphorylation are linked to cognitive decline in neurodegenerative disorders. Hyperphosphorylated Tau accumulates into toxic neurofibrillary tangles, one of the central hallmarks of AD, Chronic Traumatic Encephalopathy, and other neurodegenerative diseases (Ballatore et al. 2007). While the direct effects of Tau on neuronal dysfunction have been extensively characterized, far less is understood about how secondary factors, including diet, modulate Tau-driven neurodegeneration.

*Drosophila* provides a powerful model for investigating the interactions between diet and neurodegenerative disease. Overexpression of human Tau in flies leads to synaptic dysfunction and these effects are further exacerbated by hyperphosphorylation variants that are associated with neurodegenerative disease (Ali et al. 2012). The pathogenic Tau ^R406W^ mutation is associated with fronto-temporal dementia and has been widely used in *Drosophila* to model tauopathy and assess mechanisms of neurodegeneration (Wittmann et al. 2001). Similarly, exposure to a high-sugar diet (HSD) shortens lifespan, induces metabolic dysfunction, and impairs clearance of damaged neurites, recapitulating key features of obesity and diabetes (Alassaf and Akhila Rajan 2023). Despite these parallels, the interactions between Tau-mediated neurodegeneration and obesogenic diet remain poorly understood. Here, we test the effects of Tau overexpression on lifespan and behavioral performance under both standard and obesogenic dietary conditions.

We established neurodegeneration-model flies using the pan-neuronal driver nSyb-GAL4 to ectopically express either human non-mutant Tau (Tau ^WT^ ) or Tau ^R406W^ variant. Flies were housed on either standard food (STD) or an obesogenic, high-sugar diet (HSD) consisting of standard food supplemented with 30% sucrose. To compare lifespan across genotypes and dietary treatments, we first measured longevity in group-reared mated females up to three weeks of age. By three weeks, there was a significant increase in mortality in nSyb-GAL4>Tau ^R406W^ flies fed HSD compared to flies of the same genotype maintained on standard food. No deficits were observed in flies overexpressing Tau ^WT^ (nSyb-GAL4> Tau ^WT^ ) or in nSyb-GAL4/+ controls ( Fig. 1A ). These findings reveal interactions between Tau ^R406W^ and dietary stress result in reduced lifespan.

To more precisely determine the effects of neuronal Tau and diet on lifespan, we measured longevity in individually housed flies across their lifespan. Flies were placed in *Drosophila* Activity Monitoring Systems (DAMS), where infrared beam breaks were used to detect movement and infer time of death (Murakami et al. 2021). This analysis confirmed reduced lifespan in nSyb-GAL4> Tau ^R406W^ flies compared to nSyb> Tau ^WT^ or nSyb-GAL4/+ controls. However, in contrast to the group-reared assay, there was no significant effect of HSD on lifespan for flies expressing wild-type and mutant Tau (Fig 1B). Therefore, these findings suggest the effects of diet on lifespan are impacted by social interactions.

Neurodegeneration is associated with deficits in sensory motor behaviors, memory, and sleep. In *Drosophila,* neurodegeneration variants, including ectopic expression of Amyloid Beta and Tau ^R406W^ , are associated with reduced sleep (Ortiz-Vega et al. 2024; Tabuchi et al., 2015). Therefore, we sought to test the effects of diet and Tau on these phenotypes. Analysis of sleep from flies housed in the DAMS system revealed reduced sleep in control nSyb-GAL4/+ housed on HSD compared to those on standard food (Fig 1C,D). Conversely, there was no effect of diet on sleep in flies expressing Tau ^WT^ or Tau ^R406W^ . (Fig 1C,D). Furthermore, across diets, sleep was increased in Tau ^WT^ and Tau ^R406W^ compared to control flies harboring nSyb-GAL4 alone. Analysis of day and night sleep at day 11-14 highlighted distinct effects of genotype and diet, where HSD reduced both day and night sleep in nSyb-GAL4/+ with no effect on nSyb> Tau ^WT^ and nSyb> Tau ^R406W^ . However, both nSyb-GAL4> Tau ^WT^ and nSyb-GAL4> Tau ^R406W^ exhibited higher sleep duration as compared to the controls. Analysis of total daytime and nighttime activity showed distinct effects of genotype and diet where nSyb-GAL4/+ on HSD showed increased nighttime activity and decreased sleep, whereas nSyb-GAL4> Tau ^WT^ and nSyb-GAL4> Tau ^R406W^ overall showed decreased activity as compared to the controls. Overall the activity was significantly reduced in nSyb-GAL4> Tau ^R406W^ on HSD. These findings differ from a previous manuscript suggesting neuronal expression of Tau ^R406W^ results in shortened sleep (Ortiz-Vega et al. 2024). The differences in these findings could reflect that each manuscript used different neuronal drivers, reflecting an effect of timing or robustness of Tau ^R406W^ expression. It is also possible that the increase in sleep observed here may be reflective of reduced sleep quality that is exacerbated in flies expressing human Tau variants.

Motor behavior diminishes with age, and motor deficits are accelerated in neurodegenerative conditions. We subjected each genotype to a climbing assay, which revealed significant impairments in climbing performance for both Tau ^WT^ and Tau ^R406W^ compared to controls at 14 days of age. At the middle timepoint of the assay (30 seconds), the climbing ability of Tau ^R406W^ flies was paradoxically better on a high-sugar diet, while it was significantly reduced on a HSD in Tau ^WT^ flies (Fig 1E). Therefore, HSD varied in its effect across all genotypes, having no effect on controls, impairing climbing in Tau ^WT^ and enhancing climbing in Tau ^R406W^ flies. Taken together, neuronal expression of Tau ^WT^ and Tau ^R406W^ impairs climbing performance, but the effects of HSD are distinct across the genotypes.

Aging is associated with reduced sensory function in humans and fruit flies (Son et al. 2021; Brown et al. 2024). Therefore, we sought to define the effects of Tau and diet on chemosensory behavior. We used the Proboscis Extension Reflex (PER) assay to measure responsiveness to a fructose tastant under both standard and HSD conditions (Brown et al. 2023). Flies expressing Tau ^R406W^ displayed a significantly reduced PER response compared to nSyb-GAL4 controls (Fig 1F). HSD impaired PER in nSyb-GAL4 controls, but not either experimental line ( Fig. 1F ). Therefore, overexpression of human Tau variants, but not HSD, disrupts taste sensitivity.

Previous work has shown that metabolic stress, including impaired glucose metabolism and insulin resistance, can exacerbate tau pathology and neurotoxicity suggesting that obesogenic diets may accelerate neurodegeneration (Planel et al. 2007; Alassaf and Akilah Rajan 2023). Here, we directly tested these interactions in fruit flies. Overall, we find distinct effects on both longevity and behavior depending on the assay used. These findings highlight the complexity of pathogenesis in fruit flies and indicate that pan-neuronal Tau expression may differentially affect behaviors, revealing varied sensitivities of behaviorally relevant circuits to disease. The interaction between HSD and Tau ^R406W^ expression on longevity for social, but not solitary flies, is consistent with the notion that socially housed flies consume more food, potentially contributing to the observed effects on lifespan (Li et al. 2021). It is possible that HSD-dependent changes in behavior may require higher amounts of sucrose, testing flies at a later age, or reducing sucrose in the standard food group. Therefore, these findings do not rule out the potential for high-sugar diet, or other high-calorie diets to influence sleep across conditions. Together, these results demonstrate that diet and Tau interact in highly context-dependent ways, with obesogenic conditions modifying some, but not all, Tau-induced phenotypes. These findings underscore the need for multi-modal assays to define gene–environment interactions in neurodegeneration.

## Methods

*Drosophila* husbandry: All experiments were conducted in *Drosophila melanogaster* . Flies were reared on standard cornmeal–molasses–yeast medium prepared in-facility (1L of food, 914.3 mL water, 86.3 mL molasses, 35.5 g yeast, 62.1 g cornmeal, and 10.4 g agar were used, with an additional 215.6 mL water to dissolve yeast and cornmeal; 20% tegosept (12.8 mL) and propionic acid (6.9 mL) were added after cooling). Flies were maintained in temperature-controlled incubators (Powers Scientific, Warminster, PA, USA) at 25 °C under a 12 h:12 h light-dark (LD) cycle, with humidity maintained at 55–65%. Standard husbandry practices were followed throughout. Stocks were maintained in uncrowded vials and transferred to fresh food every 2–3 days to prevent overcrowding and nutritional depletion. Unless otherwise specified, all experiments were performed using mated females by maintaining them for at least 48 h after eclosion. For aging experiments, flies were kept on standard food or obesogenic diet and transferred to fresh vials every other day until reaching the indicated experimental age. For sucrose supplementation experiments, flies were maintained on standard food supplemented with 30% sucrose unless otherwise noted.

Fly Stocks: The wild-type fly line used in this study was *w ^1118^* (Bloomington Drosophila Stock Center, stock #5905) (Levis et al. 1985). *Tau ^WT^* (BDSC #51363), *Tau ^R406W^* (Flybase Report Fbal0126527, received from Moulton Lab), *nSyb-GAL4* (BDSC #51635). For the crosses, the virgins were collected from *Tau ^R406W^ , Tau ^WT^ , and w ^1118^* . Unless otherwise indicated, experimental manipulations and comparisons were performed relative to this stock. Stocks were expanded and maintained for at least two generations in our laboratory before inclusion in experiments, ensuring adaptation to our husbandry conditions.

Sleep Behavior & Longevity Assay: Sleep behavior was measured using the Drosophila Activity Monitoring System (DAM2; TriKinetics Inc., Waltham MA), which detects locomotor activity based on infrared beam crossings of individual flies. Flies were briefly anesthetized with CO₂ and loaded into 65 mm × 5 mm locomotor tubes containing standard Drosophila medium as mentioned above or the specified dietary intervention (e.g., sucrose-supplemented food). Flies were acclimated for a minimum of 24 hours prior to the start of behavioral analysis. Food filled at one end of each tube and was sealed with cotton plugs. Unless otherwise noted, all experiments were performed with 3–5-day-old mated female flies, which were selected to minimize age and sex-dependent variability in sleep behavior. For lifespan studies, sleep was assayed across lifespan, with flies maintained on the specific diet and transferred to fresh tubes every 5 days to prevent food desiccation and microbial contamination. Sleep profile was quantified from infrared beam crossings of individual flies. Raw activity files were retrieved using DAMFileScan (TriKinetics), and custom Python scripts were used to bin activity at 5-min resolution. Sleep was defined as periods of immobility lasting ≥5 min, and sleep traits—including total sleep, mean bout length, bout number, and waking activity, were extracted using the Drosophila Sleep Counting Macro.

Climbing Assay: Climbing ability was quantified as previously described using a negative geotaxis assay (Madabattula et al. 2015). Behavioral analysis was conducted at 14 days of age between ZT3-6. Each trial consisted of a group of 10 flies, and the average of this group was used for each data point. Flies were transferred into empty, clean plastic vials marked with an 8 cm reference line and allowed to acclimate for one minute before testing began. To initiate testing, vials were tapped sharply to bring all flies to the bottom and the number of flies crossing the 8 cm mark was recorded in 5-second intervals over an observation period of one minute. Multiple groups with the same genotype and diet were tested, and the average climbing performance was calculated along with mortality for comparison between genotypes and diet conditions.

Proboscis Extension Reflex Assay: Groups of flies maintained on standard food were either kept on a standard diet or transferred to a 30% extra sucrose diet from day 3 for 7 days before testing, followed by a starvation period of approximately 16 hours with only access to water. Starved flies were briefly anesthetized using CO2 and gently glued to glass slides as previously described in Brown et al (Brown et al. 2023). Mounted flies were then placed in a humidified chamber for 1-2 hours to fully recover. The PER assay was performed by presenting 50 mM fructose to each fly and scoring any proboscis extensions as positive responses.

Statistical Analysis: All statistical analyses were performed using GraphPad Prism (v10.6.1). Survival curves for grouped, and individually housed flies in Drosophila Activity Monitors ( Fig. 1A- B) were analyzed using the Log-rank Mantel–Cox test to test the effect of genotype and dietary condition on survival. Longevity was further tested using the Gehan–Breslow–Wilcoxon test. Pairwise comparisons between dietary conditions within each genotype were performed using Holm–Šídák’s multiple comparisons test. Sleep across lifespan, quantification at day 14 as well as the climbing assay ( Figure 1C- E) was analyzed using two-way ANOVA with genotype and dietary condition as factors. For the PER assay, all post-hoc analyses were performed using Tukey’s multiple comparisons test. All data are presented as mean ± SE. The letters shown above the bars represent compact letter displays generated from the post hoc multiple-comparison test following ANOVA. Groups that share at least one letter are not significantly different from one another. Groups with different letters are significantly different.
